# Chromosomal Aberrations and Oxidative Stress in Psoriatic Patients with and without Metabolic Syndrome

**DOI:** 10.3390/metabo12080688

**Published:** 2022-07-26

**Authors:** Drahomira Holmannova, Pavel Borsky, Ctirad Andrys, Kvetoslava Hamakova, Eva Cermakova, Gabriela Poctova, Zdenek Fiala, Jindra Smejkalova, Vladimir Blaha, Lenka Borska

**Affiliations:** 1 Institute of Preventive Medicine, Faculty of Medicine in Hradec Kralove, Charles University, 500 03 Hradec Kralove, Czech Republic; holmd9ar@lfhk.cuni.cz (D.H.); poctovag@lfhk.cuni.cz (G.P.); fiala@lfhk.cuni.cz (Z.F.); smejkal@lfhk.cuni.cz (J.S.); borka@lfhk.cuni.cz (L.B.); 2 Department of Clinical Immunology and Allergology, Faculty of Medicine in Hradec Kralove, Charles University, 500 03 Hradec Kralove, Czech Republic; andrys@lfhk.cuni.cz; 3 Clinic of Dermal and Venereal Diseases, Faculty of Medicine in Hradec Kralove, University Hospital Hradec Kralove, 500 05 Hradec Kralove, Czech Republic; kveta@hamakova.cz; 4 Department of Medical Biophysics, Medical Faculty, Charles University, 500 03 Hradec Kralove, Czech Republic; cermakovae@lfhk.cuni.cz; 5 3rd Department of Internal Medicine—Metabolic Care and Gerontology, Medical Faculty, Charles University, 500 03 Hradec Kralove, Czech Republic; blaha@lfhk.cuni.cz

**Keywords:** chromosomal aberration, psoriasis, metabolic syndrome

## Abstract

Psoriasis and metabolic syndrome (MetS), a common comorbidity of psoriasis, are associated with mild chronic systemic inflammation that increases oxidative stress and causes cell and tissue damage. At the cellular level, chromosomal and DNA damage has been documented, thus confirming their genotoxic effect. The main objective of our study was to show the genotoxic potential of chronic inflammation and determine whether the presence of both pathologies increases chromosomal damage compared to psoriasis alone and to evaluate whether there are correlations between selected parameters and chromosomal aberrations in patients with psoriasis and MetS psoriasis. Clinical examination (PASI score and MetS diagnostics according to National Cholesterol Education Expert Panel on Detection, Evaluation, and Treatment of High Blood Cholesterol in Adults; NCE/ATPIII criteria), biochemical analysis of blood samples (fasting glucose, total cholesterol, low density and high density lipoproteins; LDL, HDL, non-HDL, and triglycerides;TAG), DNA/RNA oxidative damage, and chromosomal aberration test were performed in 41 participants (20 patients with psoriasis without MetS and 21 with MetS and psoriasis). Our results showed that patients with psoriasis without metabolic syndrome (nonMetS) and psoriasis and MetS had a higher rate of chromosomal aberrations than the healthy population for which the limit of spontaneous, natural aberration was <2%. No significant differences in the aberration rate were found between the groups. However, a higher aberration rate (higher than 10%) and four numerical aberrations were documented only in the MetS group. We found no correlations between the number of chromosomal aberrations and the parameters tested except for the correlation between aberrations and HDL levels in nonMetS patients (rho 0.44; *p* < 0.02). Interestingly, in the MetS group, a higher number of chromosomal aberrations was documented in non-smokers compared to smokers. Data from our current study revealed an increased number of chromosomal aberrations in patients with psoriasis and MetS compared to the healthy population, especially in psoriasis with MetS, which could increase the genotoxic effect of inflammation and the risk of genomic instability, thus increasing the risk of carcinogenesis.

## 1. Introduction

Psoriasis is a complex chronic systemic immune-mediated disease in which a variety of exogenous and endogenous stimuli stimulate an exaggerated immune response in genetically predisposed individuals [1,2]. The disease has a bimodal distribution. A major peak occurs between the ages of 20 and 30 years, and a minor peak occurs between the ages of 50 and 60 years.

Psoriasis is associated with intermittent remissions and exacerbations caused by trigger factors such as infection, trauma, alcohol consumption, smoking, food, drugs, emotional stress, etc. [3]. Psoriasis preferentially affects the skin and usually presents as well-defined erythematous indurated plaques covered with silvery scales, epidermal hyperproliferation, increased differentiation, and keratinocyte apoptosis and neoangiogenesis. Furthermore, psoriatic inflammation also affects the nails, joints, and eyes and is associated with numerous comorbidities such as metabolic syndrome, diabetes, cardiovascular disease, malignancy including leukemia and lymphoma, etc., which have a severe negative impact on the quality and length of life of patients [4,5,6,7,8].

Metabolic syndrome is a pathological condition, a set of symptoms that predispose to the development of cardiovascular disease, diabetes mellitus, liver damage, cancer, chronic lung disease, malignancies, and other inflammatory and autoimmune diseases, including psoriasis. Metabolic syndrome is diagnosed when three of the five criteria are present: increased levels of triglycerides, increased blood pressure and waist circumference, glucose intolerance, and reduced levels of HDL (details in the section Material and Methods). The prevalence of the metabolic syndrome is highest in the elderly population and continues to increase in younger individuals, including children [9,10].

The main controllable causes of metabolic syndrome include increased food intake and decreased physical activity, leading to hypertrophy of white adipose tissue and changes in lipid and glucose metabolism. Adipose tissue is the source of various adipokines that regulate food intake, central and autonomic nervous functions, and immune system functions (e.g., leptin, adiponectin, resistin, visfatin, and tumor necrosis factor alfa; TNF-α). In the reactivity of the immune system, there is a shift to proinflammatory activity [11]. Typically, there is increased production of proinflammatory cytokines (e.g., IL-1β, IL-6, and TNF-α), infiltration of adipose tissue by immune cells (macrophages, neutrophils, and NK cells), differentiation of macrophages from subset M2 to M1 subset, increase in the number of Th17 cells, decrease in the number of regulatory T and B lymphocytes, and many additional changes. In common with psoriasis, the metabolic syndrome is associated with chronic inflammation, metainflammation [7].

Chronic inflammation exacerbates immune system imbalances and tissue damage and may increase the risk of developing other inflammatory diseases.

One of the changes in immune system reactivity that links psoriasis and metabolic syndrome is the increase in Th17 activity [12,13]. Furthermore, psoriasis is very often associated with dyslipidemia, higher levels of triglycerides, and lower levels of HDL (two criteria for MetS diagnosis) [14].

In addition to inflammation, both pathologies are associated with increased oxidative stress and the release of endogenous substances that can be involved in DNA damage [15,16,17]. Evidence of oxidative damage to DNA and RNA is the presence of oxidized nucleobases, especially guanine (8-oxo-2′-deoxyguanosine or 8-hydroxy-2′-deoxyguanosine) that is associated with DNA breaks and impaired DNA repair [18].

DNA damage includes not only changes at the nucleotide level but also damage at the chromosomal level and chromosomal aberrations. They may occur due to exposure to oxidative stress and endogenous and exogenous factors (chemicals, drugs, toxins, ultraviolet light, and ionizing radiation), and even spontaneously in healthy persons [19].

Chromosomal aberrations are classified into two basic groups: structural and numerical [20]. Structural chromosomal abnormalities are the result of chromosome breakage and incorrect joining of chromosomal segments and include chromosome rearrangements and gene amplification. However, there are also spontaneous structural rearrangements that result from recombination errors during cell division. Numerical chromosomal aberrations arise as a consequence of impaired cell and chromosome division due to disruption of the mitotic spindle apparatus [21]. To investigate the presence of chromosomal aberrations, the chromosomal aberration test (CAT) is widely used [22]. Chromosomal aberrations are more likely to be deleterious than beneficial, thus increasing the risk of cancer, recurrent abortions, accelerated aging, and degenerative disorders or inducing immune dysfunction [20,23].

Our study was carried out to analyze the rate of chromosomal aberrations in peripheral blood mononuclear immune cells from patients with psoriasis with or without metabolic syndrome (MetS and nonMetS, respectively). We wanted to determine whether inflammation associated with psoriasis and the combination of psoriasis and MetS has genotoxic potential and increases the DNA/RNA damage and the frequency of chromosomal aberrations. MetS is a very common comorbidity of psoriasis and its incidence and prevalence is increasing in the general population, including among psoriatic patients, in whom it may lead to more severe health complications compared to otherwise healthy persons.

We did not find any other study to address this issue, so this is the first study to explore the genotoxic effect of the combination of psoriasis and MetS.

## 2. Materials and Methods

### 2.1. Patients

The study enrolled 41 patients with psoriasis (mild to moderate according to PASI score) who were investigated at the Clinic of Dermal and Venereal Disease, Charles University Hospital in Hradec Kralove. They were divided into two subgroups: patients with metabolic syndrome (MetS; number = 21) and patients without metabolic syndrome (nonMetS; n = 20). Patients with acute or chronic inflammatory diseases, malignancies, or pregnancy and those taking non-steroidal or anti-inflammatory medications were excluded from the study. Patients with psoriasis had no form of psoriasis treatment three months prior to the study. The study was carried out according to the Declaration of Helsinki and the protocol was approved by the Ethics Committee of the Charles University Hospital in Hradec Kralove, Czech Republic.

### 2.2. PASI

For a standardized clinical evaluation, Psoriasis Area Severity Index (PASI) was used. The severity is calculated based on erythema, desquamation, and skin infiltration to assess the severity of psoriasis [24].

### 2.3. Metabolic Syndrome

The diagnosis of metabolic syndrome was determined according to the criteria of the National Cholesterol Education Program Adult Treatment Panel (NCE/ATPIII) when the presence of three of the five listed criteria is confirmed [25].

- Increased waist circumference (abdominal obesity ≥102 cm for men and ≥88 cm for women);
- Glucose intolerance presented by a higher fasting glucose of 5.6 mmol/L or known treatment for diabetes;
- Elevated triglyceride level (TAG) 1.7 mmol/L;
- Reduced level of high-density lipoproteins (HDL cholesterol) <1.03 mmol/L for men and <1.30 mmol/L for women;
- Elevated blood pressure (systolic blood pressure < 130 mmHg and/or diastolic blood pressure < 85 mmHg).

Waist circumference, systolic and diastolic blood pressure, and PASI were evaluated at the Clinic of Dermal and Veneral Diseases, Charles University Hospital and Faculty in Hradec Kralove.

### 2.4. Blood Sampling

#### 2.4.1. Biochemical Parameters

Fasting glycemia, triacylglycerol, cholesterol, HDL, LDL, and non-HDL were measured in serum from blood samples withdrawn from the cubital vein using standard laboratory methods at the Institute of Clinical Biochemistry and Diagnostics (FN and LF UK in Hradec Kralove).

#### 2.4.2. Oxidative Damage to DNA/RNA

Blood samples collected from the cubital vein (by BD Vacutainer sampling tubes) were centrifuged, and isolated blood serum was stored at −70 °C until analysis. DNA/RNA oxidative damage was evaluated using the EIA Kit (Enzyme Immunoassay, Cayman Chemical Company, Ann Arbor, MI, USA) according to the manufacturer’s instructions. Damage was assessed as the sum of three oxidized guanines in serum: 8-hydroxy-2′-deoxyguanosine, 8-hydroxyguanosine, and 8-hydroxyguanine, with a detection limit of 33 pg/mL.

#### 2.4.3. CAT

Blood samples were collected from the cubital vein into a vacuum tube with lithium heparin (VACUETTE^®^ TUBE, 4 mL LH Lithium Heparin, Greiner Bio-One GmbH, Austria). The samples were stored in a refrigerator at a temperature between +4 to +7 °C and processed within 24 h after sampling. The next steps of CAT were performed according to the protocol available at https://dx.doi.org/10.17504/protocols.io.mjgc4jw (accessed on 15 June 2022). (Malkova, Tichy). In each sample, 100 mitotic cells were microscopically evaluated and the total number of aberrated cells and the number of structurally and numerically aberrated cells were determined (AHEM 1/2007) [26,27].

#### 2.4.4. Group Analysis

We analyzed the rate of chromosomal aberration and compared with guidelines (AHEM 1/2007).

<2% total number of aberrated cells (ABB), the value identical to the spontaneous frequency of aberrations in a normal and healthy population.

2–4% ABB indicates increased exposure to genotoxic (endo or exogenous) substances and factors that the organism cannot tolerate.

>4% ABB indicates high exposure to genotoxic substances or factors. 

#### 2.4.5. Individual Analysis

ABB of >5% indicates high exposure to genotoxic substances and factors or reduced efficiency of reparative or other mechanisms.

### 2.5. Statistical Analysis

Quantitative data are presented by median, 1st and 3rd quartiles, minimum, and maximum. The two-sample *t*-test and nonparametric Mann–Whitney test were used to compare groups. Spearman rank correlations were used to evaluate the relationship between the parameters. Qualitative data are presented by counts and percentages, and Fisher’s exact test was used. Data are graphically presented by box and scatter plots. The level of significance α < 0.05 was considered statistically significant. Statistical analysis was performed using statistical software NCSS 2021 Statistical Software (2021). (NCSS, LLC., Kaysville, UT, USA, https://ncss.com/software/ncss, accessed on 15 June 2022).

## 3. Results

### 3.1. Demographic Data of Participants

The group of 41 participants included 21 patients with psoriasis with metabolic syndrome (MetS) and 20 patients without MetS (nonMetS). The distribution of age (Mets median 55 and nonMets median 51.9 years), sex (MetS 10 women and 11 men; nonMetS 9 women and 11 men), and smoking habits (MetS 12 non-smokers and 9 smokers; nonMetS 13 non-smokers and 7 smokers) did not differ significantly between the groups (Table 1). As expected, some parameters that reflect the presence of metabolic syndrome were significantly higher in the MetS group: fasting glucose (*p* value > 0.02), high-density lipoprotein (HDL; *p* < 0.001), triglycerides (TAG; 0.001), body mass index (BMI; *p* < 0.0001), waist circumference (*p* < 0.001), and systolic blood pressure (*p* < 0.01) (Table 2).

### 3.2. DNA/RNA Damage

**Figure 1:**
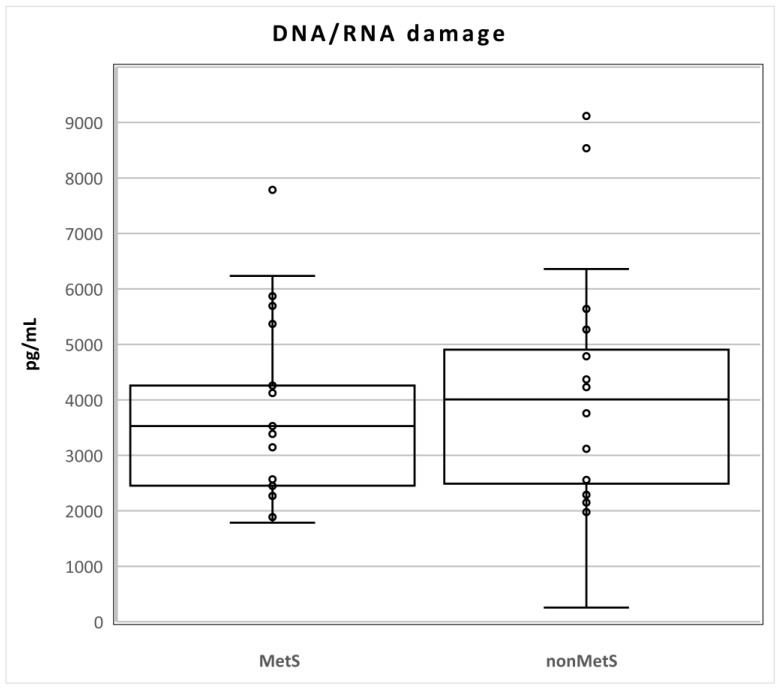
Levels of DNA/RNA damage in MetS and nonMetS patients. Legend: The horizontal line in the boxes indicates the position of the median, the ends of the boxes define the 25th and 75th percentiles, and error bars mark the 10th and 90th percentiles. Y is an outlier.

The levels of DNA/RNA damage did not differ statistically between the two groups of patients (Mets n = 21; median 3528, Q1–Q3 2451–4814; nonMets, median 4009, Q1–Q3 2356–5148; pg/mL; *p* = 0.611; Figure 1). 

### 3.3. Chromosomal Aberrations

**Figure 2:**
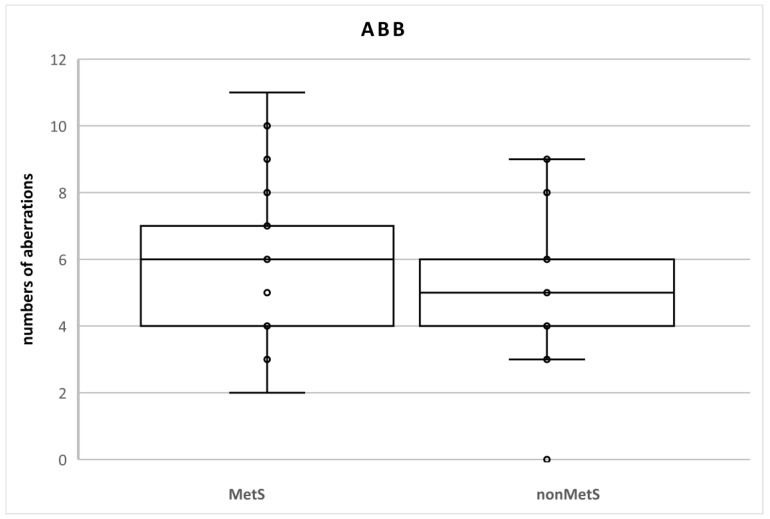
Numbers of chromosomal aberrations in MetS and nonMetS patients. Legend: The horizontal line in the boxes indicates the position of the median, the ends of the boxes define the 25th and 75th percentiles, and error bars mark the 10th and 90th percentiles. Y is an outlier.

There were no statistical differences between the groups of patients (Table 2). We identified 120 aberrations in the total number of 2100 cells (100 cells in each sample; n = 21; 5.7%) in MetS patients and 105 aberrations in the total number of 2000 cells (100 cells in each sample; n = 20; 5.3%) in nonMetS patients. The SAB levels were 116 and 105 (5.5% and 5.3%, respectively) and NAB levels were 4 and 0 (0.2% and 0%, respectively) (Table 3, Figure 2).

At individual levels (number of aberrations in one sample = 100 mitotic cells), a rate of ABB ≥ 5% was found in 8 (40%) and 11 (52.4%) nonMets and MetS patients, respectively. ABB < 10% was detected in only two patients with MetS (9.5% vs. 0%). Numerical aberrations were observed only in three MetS patients. One of them had two NAB (15% vs. 0%) (Table 4).

**Figure 3:**
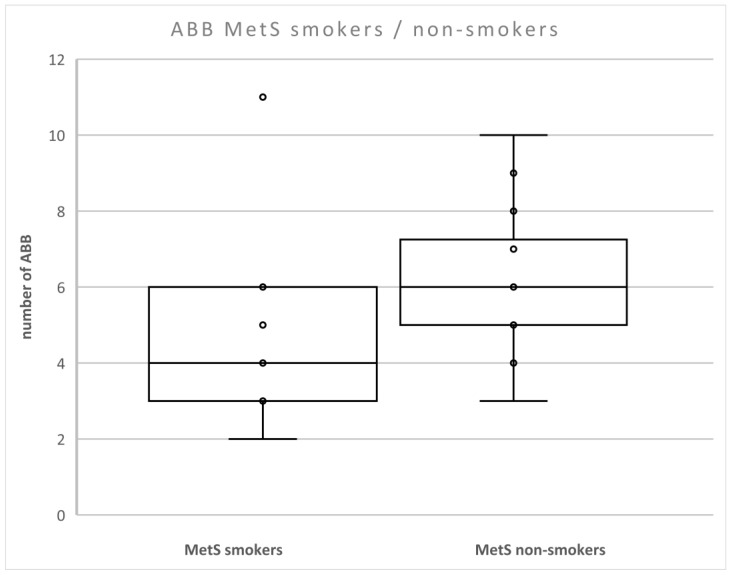
Number of aberrations in smokers and non-smokers with MetS. Legend: The horizontal line in the boxes indicates the position of the median, the ends of the boxes define the 25th and 75th percentiles, and error bars mark the 10th and 90th percentiles. Y is an outlier.

We found that the level of ABB was lower in patients with MetS compared to non-smokers with MetS (*p* < 0.05) (Figure 3). There were no differences between smokers and non-smokers in nonMetS patients.

### 3.4. Relationships among the Evaluated Parameters

**Figure 4:**
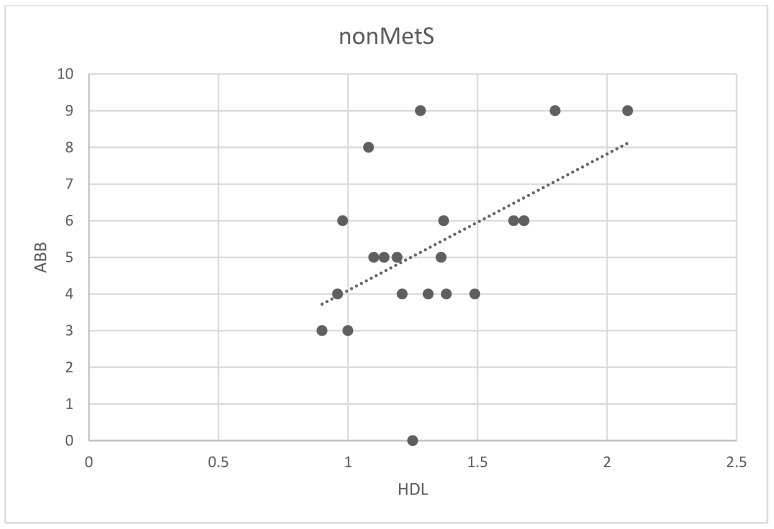
Correlation between the number of ABB and HDL levels in non-MetS patients. Legend: The scatter plot shows the relationship between the number of ABB and HDL levels. Spots represent the plotted values of the measured variables obtained for each patient, and the line represents the best fit for the correlation.

We analyzed possible associations among ABB and all analyzed parameters in the main group and two subgroups (all patients, patients nonMetS, and patients MetS). The only correlation found was between ABB and HDL in the nonMetS group (Spearman correlation 0.44; *p* < 0.02, Figure 4). 

## 4. Discussion

Psoriasis and metabolic syndrome are chronic systemic inflammatory diseases that are increasing in incidence, especially metabolic syndrome. Our study focused on the genotoxic effect of these pathologies that manifests as chromosomal aberrations. The study population consisted of 41 patients with psoriasis: 21 with MetS and 20 without MetS.

As expected, patients with MetS had elevated values of parameters that serve as criteria for the diagnosis of MetS (fasting glucose, HDL, TAG, BMI, waist circumference, and systolic blood pressure). None of these parameters correlated with chromosomal aberrations except HDL in nonMetS patients. There are no such results in the literature. Our explanation is therefore based on the available information on HDL functions.

Although HDL is known primarily as a factor that protects the cardiovascular system from damage caused by cholesterol deposition in blood vessel walls and tissues and has anti-inflammatory effects, studies have shown that elevated HDL levels may in turn be pro-inflammatory and pro-atherogenic [28,29,30].

There is evidence that although patients with psoriasis are more likely to have reduced HDL levels, in some cases, HDL levels may be elevated and the combination of HDL and inflammation may alter normal HDL functions and enhance its detrimental activity [31].

We should also take into account the fact that patients with metabolic syndrome are taking drugs such as antihypertensives or hypolipidemics, which may interfere with inflammatory processes and the effect of HDL, thereby reducing its possible negative influence on the development of chromosomal abnormalities [32,33]. Therefore, there is the possibility that in patients with psoriasis, HDL may be a damaging factor while in patients with psoriasis and MetS, it may have protective or neutral functions due to the treatment administered.

Even mild inflammatory processes are associated with tissue damage and increased production of reactive oxygen species, which are responsible for damage to molecules, including DNA and RNA. Being aware of these data, we analyzed oxidative damage to genetic material. Oxidative DNA/RNA damage is reflected in nucleobase oxidation, mainly guanine and/or chromosomal abnormalities [34,35].

In our previous studies, we have shown that psoriasis is associated with increased inflammation compared to the healthy population, which can be slightly amplified by the presence of MetS. Levels of DNA/RNA damage were elevated in psoriasis patients compared to healthy controls. Furthermore, DNA/RNA damage levels were slightly higher in patients with MetS than in psoriatic patients [36,37]. In this study, we found no difference in DNA/RNA damage between patients with MetS and nonMetS patients. Furthermore, no association was found between DNA/RNA damage and chromosomal aberrations. We hypothesized that this correlation may occur primarily in the MetS group. The association between DNA/RNA damage and chromosomal aberration has been documented. Usman et al. confirmed that oxidative DNA damage in obese children is a predictor of genomic instability [38,39].

The inflammatory state, together with an increase in oxidative stress, may have a genotoxic effect. The prevalence of chromosomal aberrations was higher in patients in our study than in the general population, where the population limit is up to 2%. 

Interestingly, the presence of two pro-inflammatory pathologies did not statistically increase the number of total chromosomal aberrations (median 6% MetS and 5% nonMets). Although the result was not statistically significant, we must emphasize that the number of persons with total number of abberated cells (ABB) > 5% was higher in the individuals from the MetS group (40% vs. 55.4%). Moreover, ABB ≥ 10% was found only in two patients with MetS, as well as numerical aberrations, which were found in three individuals with MetS. One patient with MetS had two numerically aberrated cells. No numerical aberrations were detected in nonMetS patients.

We did not document the correlation between aberrations and other measured parameters and except the above-mentioned HDL.

Other studies, like ours, have shown that psoriasis is associated with chromosomal instability. Karaman et al. evaluated the exchange of DNA fragments between sister chromatids (SCE) in patients with psoriasis and compared the results with healthy controls. They discovered that psoriasis increased the rate of SCE [40]. Molès et al. revealed that psoriasis increased the amount of cytosolic DNA in keratinocytes. The formation of cytosolic DNA is dependent on the breakdown of DNA. Furthermore, they detected cytosolic RNA:DNA duplexes [41]. Impaired DNA repair is involved in chromosomal instability. Rodríguez-Jiménez found that patients with psoriasis had a reduced expression of GADD45a compared to healthy individuals. GADD45a is an important player in the processing of DNA repair [42]. Although these studies have suggested that psoriasis has a genotoxic effect, Ranna et al. and Malkova et al. did not detect any or low levels of chromosomal aberration in persons with psoriasis [43,44].

Not only psoriasis can be accompanied by DNA damage. MetS is also associated with chromosomal damage. As mentioned above, only persons with MetS in our study had both structural and numerical aberrations, and two patients had ≥ 10% aberrated cells. Although the differences in values were not statistically significant between the nonMetS and MetS groups, it is evident that MetS is an aggravating factor in patients in terms of DNA damage. The DNA damaging effect of MetS, obesity, diabetes, etc., was described by Dasouki et al. in their review [45]. Chromosomal damage in persons with diabetes and obesity was discussed by Franzke et al. in their review with a meta-analysis [46]. Anand et al. suggested that diabetes is a risk factor for cancer, precisely due to the increased incidence of chromosomal aberrations [47]. Bankoglu et al. demonstrated that obesity is directly related to DNA damage since bariatric surgery with weight reduction reduced chromosomal damage [48]. Importantly, Fieres et al. described the association between BMI and a decrease in DNA repair activity [49]. Thus, all these studies show that MetS is a risk factor for DNA damage and genomic instability.

Both pathological conditions, psoriasis and MetS, are associated with the risk of cancer. Malignant transformation may be caused by the aforementioned chromosomal damage and genomic instability that can be induced by chronic inflammation and oxidative stress or immunosuppressive therapy for severe psoriasis. Nagel et al. evaluated the association of different blood cancers and MetS in 578,000 adults. In particular, BMI was positively correlated with the risk of blood cancer (lymphoid neoplasms and Hodgkin lymphoma in women and high-grade B-cell lymphoma and chronic lymphatic leukemia in men and women). Importantly, the MetS score was associated with a 48% increased risk of Hodgkin lymphoma in women [50]. MetS also increases the risk of cervical, ovarian, liver, colorectal, and pancreatic cancer [51]. The meta-analysis by Vaengebjerg et al. analyzed data from 122 studies (n  =  2,053,932 patients). They found an increased risk of keratinocyte cancer and lymphomas [52]. The association between an increased risk of lymphomas confirmed the study by Fuxench et al. who documented that the adjusted hazard ratio for lymphoma was 1.34, 1.31, and 1.89 according to the severity of the disease. They also described a higher risk for lung cancer and non-melanoma skin cancer [53]. The meta-analysis by Bellinato et al. showed a strong association between psoriasis and lymphohematological malignancies, such as Hodgkin and non-Hodgkin lymphoma, multiple myeloma, and leukemia [54]. Furthermore, the presence of psoriasis and MetS (chronic inflammation) can influence biological aging. Both pathologies are associated with an acceleration of biological aging. Psoriasis affects aging mainly in women [55,56].

Gheucă-Solovăstru et al. and Traffold et al. in their meta-analysis confirmed the association between psoriasis and various types of cancer, including the central nervous system, digestive tract (upper and lower), bladder, and lung cancer [57,58]. Our results demonstrating an increased incidence of chromosomal aberrations in people with psoriasis and people with psoriasis and MetS compared to the general population suggest a pro-carcinogenic potential of these diseases. 

Inflammation, DNA damage, and aberrations are also connected to smoking [59,60,61]. Both groups of patients also included smokers; therefore, we tested the association between smoking and ABB. Although the available data suggested that smoking may be an aggravating and exacerbating factor that may increase the number of ABBs, the number of ABBs was higher in the group of non-smokers with MetS compared to smokers with MetS. Smoking habits in the non-Mets group were not related to ABB. These results contradict our hypothesis and several studies [62,63]. However, existing studies describe an anti-inflammatory effect of smoking. Nicotine can reduce obesity-related inflammation by restoring glucose homeostasis and insulin sensitivity, have an immunosuppressive effect, and reduce ROS release [64,65]. However, due to the limited number of participants in subgroup analyses and studies addressing this problem, it is not possible to draw firm conclusions about the positive or protective effects of smoking in patients with MetS.

There is no study that compares the presence of aberrations in patients with psoriasis and patients with MetS. In our previous studies and this current study, there has been a noticeable pattern in responses to the presence of both pathologies in patients. Although not always statistically significant, the levels of markers that reflect immune system activity and aberrations are higher in patients with MetS compared to patients with psoriasis alone. This could suggest that the combination of psoriasis and MetS is riskier for the development of certain comorbidities, such as cancer or cardiovascular or other inflammatory diseases, especially with long-term disease duration. Early diagnosis of psoriasis and its treatment is extremely important to avoid complications, including the development of metabolic syndrome. Treating physicians should be aware of the risk of metabolic syndrome in people with psoriasis. They should intervene prophylactically and educate patients on how the development of metabolic syndrome can be prevented. If it already has occurred, it should be treated.

The study limitations include the relatively low sample size of 41 patients with psoriasis due to strict inclusion criteria, geographical localization of central Europe, solely Caucasian population, and the need to compare the results to population-based data from a database. 

## 5. Conclusions

Our study did not confirm that MetS increased oxidative DNA/RNA damage but showed that the rate of chromosomal aberrations in patients with psoriasis with and without metabolic syndrome was higher compared to the healthy population, and based on the results, we might suggest that psoriasis in combination with metabolic syndrome may cause more severe chromosomal damage. In the absence of studies that evaluate chromosomal damage in patients with psoriasis in combination with MetS, our study is very important because one of the most common comorbidities of psoriasis is MetS, whose incidence and prevalence continues to increase across the population and among psoriasis patients. Especially in patients with psoriasis who already have chronic inflammation, MetS can have much more serious consequences (cardiovascular disease or cancer) and therefore, MetS needs to be correctly diagnosed and treated.

## Figures and Tables

**Table 1:** Biochemical parameters and parameters associated with MetS and psoriasis.

Measured Parameters	Mets; n = 21; nonMetS; n = 20	Median	Q1–Q3	*p* Value
Glu mmol/L	MetS	5.1	4.53–6.91	<0.02
	nonMetS	4.48	3.7–4.97	
Chol mmol/L	MetS	4.7	4.23–5.49	NS; *p* = 0.875
	nonMetS	4.77	4.2–5.45	
HDL mmol/L	MetS	0.91	0.83–1.05	<0.001
	nonMetS	1.27	1.09–1.46	
TAG (mmol/L)	MetS	1.92	1.75–2.66	<0.001
	nonMetS	1.01	0.9–1.46	
LDL (mmol/L)	MetS (n = 20)	2.64	2.24–3.47	NS; *p* = 0.738
	nonMetS	2.92	2.17–3.5	
BMI	MetS	30.5	28.1–32.2	<0.001
	nonMetS	24.75	24.3–28.45	
Waist (cm)	MetS	103	98–111	<0.001
	nonMetS	88.5	84–98	
sBP (mmHg)	MetS	140	130–150	<0.01
	nonMetS	130	121–140	
dBP (mmHg)	MetS	90	88–100	NS; *p* = 0.203
	nonMetS	90	81–95	
PASI	MetS	15.6	13.2–30.5	NS; *p* = 0.51
	nonMetS	14.7	12.15–20.15	
DoI (years)	MetS	8	4.5–22	NS; *p* = 0.815
	nonMetS	10	6.25–19.5	

Legend: Glu, fasting glucose; Chol, total cholesterol; nonHDL, non-high-density lipoprotein; LDL, low-density lipoprotein, BMI, body mass index; sBP, systolic blood pressure; dSB, diastolic blood pressure; DoI, duration of illness; NS: statistically nonsignificant.

**Table 2:** Group analysis; the total number of aberrated cells in all samples.

Numbers of Analyzed Cells	ABB	SAB	NAB
MetS (2100 cells)	120 (5.7%)	116 (5.5%)	4 (0.2%)
nonMetS (2000 cells)	105 (5.3%)	105 (5.3%)	0

Legend: ABB, the total number of aberrated cells; SAB, structural aberrations; NAB, numerical aberrations.

**Table 3:** Numbers of chromosomal aberrations in MetS and nonMetS patients.

Numbers of Patients	ABB			SAB			NAB	
n = 41	Median	Q1–Q3	Min, Max	Median	Q1–Q3	Min, Max	Total Number	*p*-Value
MetS (n = 21)	6	4–7	2, 11	5	4–7	2, 11	4	NS, *p* = 0.70
nonMetS (n = 20)	5	4–6	0, 9	5	4–6	0, 9	0	

Legend: ABB, the total number of aberrated cells; SAB, structural aberrations; NAB, numerical aberrations.

**Table 4:** Individual analysis, number of persons with aberration.

Percentage of Total Aberration	nonMetS n = 20	MetS n = 21
≥5% ABB	8 (40%)	11 (52.4%)
≥10% ABB	0 (0%)	2 (9.5%)
NAB	0 (0%)	3 (19%) (1 person had 2 aberration)

## Data Availability

The data supporting this article are available upon request to the corresponding author. The data are not publicly available due to maintaining a high level of privacy for the study subjects.
